# Arguing in Favor of Revising the *Simulator Sickness Questionnaire* Factor Structure When Assessing Side Effects Induced by Immersions in Virtual Reality

**DOI:** 10.3389/fpsyt.2021.739742

**Published:** 2021-11-05

**Authors:** Stéphane Bouchard, Maxine Berthiaume, Geneviève Robillard, Hélène Forget, Camille Daudelin-Peltier, Patrice Renaud, Caroline Blais, Daniel Fiset

**Affiliations:** ^1^Département de Psychoéducation et de Psychologie, Université du Québec en Outaouais (UQO), Gatineau, QC, Canada; ^2^School of Psychology, University of Ottawa, Ottawa, ON, Canada; ^3^Centre de recherche du Centre Intégré de Santé et des Services Sociaux de l'Outaouais, Gatineau, QC, Canada; ^4^Innovation, Science and Economic Development Canada, Ottawa, ON, Canada

**Keywords:** simulator sickness questionnaire, simulator sickness, cybersickness, trier stress social test, anxiety, virtual reality

## Abstract

Two issues are increasingly of interest in the scientific literature regarding unwanted virtual reality (VR) induced side effects: (1) whether the latent structure of the *Simulator Sickness Questionnaire* (*SSQ*) is comprised of two or three factors, and (2) if the *SSQ* measures symptoms of anxiety that can be misattributed to unwanted negative side effects induced by immersions in VR. Study 1 was conducted with a sample of 876 participants. A confirmatory factor analysis clearly supported a two-factor model composed of nausea and oculomotor symptoms instead of the 3-factor structure observed in simulators. To tease-out symptoms of anxiety from unwanted negative side effects induced by immersions in VR, Study 2 was conducted with 88 participants who were administered the *Trier Stress Social Test* in groups without being immersed in VR. A Spearman correlation showed that 11 out of 16 side effects correlated significantly with anxiety. A factor analysis revealed that items measuring general discomfort, difficulty concentrating, sweating, nausea, and vertigo loaded significantly on the anxiety factor comprised of items from the *State-Trait Anxiety Inventory*. Finally, a multiple regression indicated that the items measuring general discomfort and difficulty concentrating significantly predicted increases in anxiety. The overall results support the notion that side effects associated with immersions in VR consist mostly of a nausea and an oculomotor latent structure and that a few items are confounding anxiety and cybersickness. The data support the suggestion to revise the scoring procedures of the *Simulator Sickness Questionnaire* when using this instrument with immersions in VR.

## Introduction

Unwanted negative side effects induced by an immersion in virtual reality (VR) are not uncommon. Cobb et al. (1) and Wilson (2) summarized the results of a comprehensive research program conducted on 148 civilians and 75 non-civilians in VR. Overall, they found that 20% of their civilian participants did not notice any side effects during an immersion in VR. Among the remaining participants, only a few (5% of the total sample) experienced side effects severe enough that they had to stop the immersion. The other participants reported side effects that, usually, were mild, occurred within the first 15 min of the immersion, and subsided within 10 min after the immersion. These side effects are often referred to in the popular literature as cybersickness, although people experiencing them are not actually sick. In a literature review on the topic, Lawson et al. (3) concluded that about 5% of users immersed in VR will report symptoms that are significant enough to warrant stopping the immersion, about 5% will not experience any symptoms at all, and the remaining users (between 70 and 90%) may experience some mild symptoms caused by the immersion in VR. Lawson (4) reported that between 50 and 100% of users immersed in VR experienced some dizziness and between 20 and 60% of users experienced some abdominal symptoms. The frequency of other symptoms appeared less documented but included oculomotor problems. These findings are consistent with Cobb et al. (1), Nichols and Patel (5), and Stanney and Kennedy (6). Similarly, Sharples et al. (7) found that 60 to 70% of participants reported an increase in unwanted VR-induced symptoms from pre- to post-immersion. Several more recent studies (8–11) and a systematic review (12) confirmed that many participants experience unwanted negative side effects induced by immersions in VR, even when current generation VR head-mounted displays (HMDs) are used.

According to Kennedy et al. (13), the temporary negative side effects associated with immersion in VR include general discomfort, difficulty focusing, increased salivation, sweating, fullness of head, stomach awareness, and burping and are regrouped into three factors: (1) *oculomotor problems* (i.e., eyestrain, blurred vision, headache), (2) *disorientation* (i.e., vertigo, imbalance), and (3) *nausea* (i.e., vomiting, dizziness). The ocular problems are likely related to the display system, such as wearing the HMD (e.g., the HMD is too heavy or too tightly strapped to the head) or the eyestrain of looking for a long period of time at computer monitors that are located at a closed and fixed distance from the eyes. The nausea and the disorientation side effects are temporary and often associated with motion sickness symptoms. The most often reported explanation for symptoms of nausea is a conflict between information provided by the otolith organs (linear acceleration of the head), the semicircular canals (angular acceleration of the head), the visual system (position and orientation of the body with respect to the visual environment), and the kinesthetic system (limb and body position) (14–19). The sensory conflict theory is not without its critics e.g., (20, 21) and other theories may explain some of the nausea symptoms, such as difficulty maintaining postural stability in virtual environments (VEs) (21, 22). For the disorientation symptoms, the most important symptoms, as measured by the *Simulator Sickness Questionnaire* [*SSQ*; (13)], are dizziness, blurred vision, difficulty focusing, and nausea. Lawson et al. (3) and Lawson (4) mentioned a fourth cluster of symptoms described as the Sopite syndrome. The Sopite syndrome is a form of motion sickness manifesting itself solely by signs of fatigue (drowsiness, difficulty concentrating, and apathy). It is possible that this syndrome involves the vestibular system. Factors associated with the Sopite syndrome after an immersion in VR remain poorly understood (3, 23, 24).

The *SSQ* (13), mentioned above, is the most widely used measure to assess simulator sickness and unwanted negative side effects following immersions in VR. The *SSQ* was developed following the notion that symptoms experienced in flight simulators are similar to sickness symptoms caused by traveling (“kinetosis” or “naupathia”), but tend to be less severe, to have a lower incidence (25), and to be more associated with the visual system and the atypical interaction among the visual, vestibular, and proprioceptive systems (26, 27). Sixteen symptoms are listed in the *SSQ*, and their severity is rated from “0” (“*None*”) to “3” (“*Severe*”). The *SSQ* was conceived for immersions in various simulators, including those frequently used in VR, such as HMDs and CAVE systems. The factor structure of the *SSQ* is based on Lane and Kennedy's (28) and Kennedy et al.'s (13) study on a sample of 1,119 military participants who were immersed in a variety of Navy simulator training exercises. The researchers wanted to determine which symptoms demonstrated systematic changes before and after the virtual immersions, and thus administered the *SSQ* before and after each immersion. After performing a principal factor analysis with a Varimax rotation and comparing factor solutions from three to six factors, and a hierarchical analysis to produce and validate a general measure that avoids problems of collinearity, a three-factor solution was identified: (1) *oculomotor* (i.e., general discomfort, eyestrain, difficulty concentrating, blurred vision, difficulty focusing, etc.), (2) *disorientation* (i.e., dizziness, vertigo, blurred vision, difficulty focusing, nausea, etc.), and (3) *nausea* (i.e., nausea, burping, increased salivation, general discomfort, difficulty concentrating, etc.). This factor structure of the *SSQ* has been widely used since then e.g., (3, 23). Other instruments have also been developed over the years, such as the *Nausea Profile* (29) and the *Virtual Reality Symptom Questionnaire* (30), but they remain far less popular than the *SSQ*.

Two themes of discussion are emerging in the literature regarding side effects induced by immersions in VR and their assessment with the *SSQ*: (1) the factorial structure of the *SSQ*, when immersive technologies differ from flight or driving simulators, and with users from the general population, and (2) the potential confound with anxiety symptoms and those measured by the *SSQ*.

As noted by Kennedy et al. (13), many items of the *SSQ* contribute significantly to more than one factor, which is a problem according to classical factor analytic approaches (31, 32). Five items are scored on two different subscales and, following Kennedy et al.'s (13) scoring procedure, are thus scored twice in the calculation of the total score. The items “general discomfort” and “difficulty concentrating” were assigned to both the Nausea and Oculomotor subscales, the items “difficulty focusing” and “blurred vision” were assigned to both the Oculomotor and Disorientation subscales, and “nausea” was assigned to both the Nausea and the Disorientation subscales. Items scored twice are essentially given more weight to the total score than other negative unwanted side effects. Multiplying the results by a constant during the scoring process further impacts the intuitiveness of the interpretation of the results.

Given the slightly blurred factor structure of the *SSQ*, Bouchard et al. (33) suggested that a two-factor solution is probably more adequate than the three-factor solution proposed by Kennedy et al. (13). The difference in factor structure may be due to differences in the type of simulations (i.e., flight simulators vs. HMD used in the treatment of phobias) or the populations for which the *SSQ* was originally developed (i.e., physically fit military personnel vs. general public). The revised factor structure of the *SSQ* proposed by Bouchard et al. (33) questions whether disorientation represents a relevant and distinct group of symptoms, and has clinical and practical implications (34).

When using the *SSQ* in a clinical context, such as with people with mental illness or for physical rehabilitation, many researchers have also noted an overlap between symptoms of anxiety and unwanted side effects of immersions in VR e.g., (35–40). In one study, Pot-Kolder et al. (41) found that anxiety partially mediated nausea and disorientation symptoms. When using VR with clinical populations, or when inducing anxiety in VR to confront feared stimuli, anxiety may better explain symptoms such as sweating, discomfort, or fatigue than immersion in VR *per se*. Given the increasing use of VR in mental health applications, Bouchard et al. (35) studied unwanted negative side effects induced by immersions in VR in a clinical sample of 157 adults diagnosed with an anxiety disorder. They found that 80% of their participants reported no or only a few mild symptoms. Interestingly, many symptoms usually associated with immersions in VR were already present before the immersion [for an example with another clinical population, see also (42)]. This suggests that the symptoms may have been anxiety-related instead of VR-related, as participants were already experiencing them pre-immersion. However, their methodology did not allow to discriminate whether the symptoms were caused by anxiety experienced during the immersions or to the immersion *per se*. To clarify the specific role of immersions in VR and anxiety on the *SSQ* and its factors, it would be necessary to induce anxiety without immersing people in VR and assess the relationship between anxiety and the symptoms of unwanted negative side effects measured by the *SSQ*.

The current article reports on two studies questioning how the *SSQ* is currently used to assess unwanted negative side effects of immersions in VR: (1) testing the adequacy of the proposed two-factor structure of the *SSQ*, and (2) documenting the potential confound of symptoms caused by anxiety.

## Study 1

### Materials and Methods

Two research questions were addressed about the factor structure of the *SSQ*: (1) Can the three-factor structure found by Kennedy et al. (13) be replicated in a large sample of non-military adults immersed in VR? and (2) Would a factor analysis confirm the adequacy of a two-factor solution? To this end, a French-Canadian translation of the 16-item *SSQ* was used (33).

#### Participants

The convenience sample consisted of 876 adults (551 women, 324 men) recruited in previous studies from the general population and suffering from an anxiety disorder (*n* = 346), gambling disorder (*n* = 77), or healthy controls (*n* = 453) (for specific details about the sample, see the list of references in the Supplementary Material). Participants were recruited via local universities' networks, advertisements in local newspapers and on social media, and referrals from clinicians. Clinical participants received their diagnoses using a structured diagnostic interview and healthy controls were screened for the absence of anxiety disorders, gambling disorder, psychotic disorder, and schizophrenia. Among the 346 anxious participants, the most frequent diagnosis was specific phobia, followed by social phobia, generalized anxiety disorder, panic disorder with agoraphobia, post-traumatic stress disorder (PTSD), and obsessive-compulsive disorder. The mean age of the total sample was 34.88 (*SD* = 13.27, range between 15 and 76).

#### Procedures

The participants completed the *SSQ* before and immediately after immersions in VR, and only post-immersion questionnaires were used in the current study. In the case of participants immersed more than once in VR, only their first immersion was included in the statistical analyses to avoid multicollinearity. Participants were immersed in VR with different technologies (i.e., HMD, CAVE-like), in a variety of VEs (clinical participants were immersed in VEs created for the treatment of anxiety disorders or gambling disorder), had to perform different tasks (i.e., exposure to feared stimuli, exploration and navigation, attention), and were immersed for different durations (immersions lasted between 5 and 60 min). For more details about the virtual environments and the tasks that participants completed, see the list of references in the Supplementary Material. Such variety in procedures favors the generalization of the results. The UQO Research Ethics Board approved the project and participants had to remain in the waiting room 15 min after the immersions before leaving the laboratory. While in the waiting room, they received a handout describing what cybersickness is and provided with contact information in the event that they experienced after-effects or prolonged side effects after the studies.

#### Measures

*Structured Clinical Interview for DSM-IV* [*SCID-IV*; (43)]. This is a semi-structured interview used to screen all participants and diagnose mental disorders according to DSM-IV criteria (44). Although the DSM-5 (45) has been published after the start of the data collection, and the SCID-5 much later, all participants from the study listed above meet DSM-5 diagnostic criteria.

*Simulator Sickness Questionnaire* (13). The 16-item *SSQ* was used to assess participants' sickness levels before and after immersions in VR. Participants rated the severity of each symptom (e.g., dizziness, headache, sweating) on a 4-point Likert scale (0 – “*None*” to 3 – “*Severe*”). To obtain a total score, all raw items were summed (the items were not weighted with Kennedy et al.'s formula to avoid inflating total scores, as suggested by) (33). A higher total score reflects more severe sickness.

### Results

The ratio of participants per variable was 55 to 1, confirming that the sample met basic assumptions and criteria to perform confirmatory factor analyses. Only participants with no missing data on all items were included in the analyses (nine participants, or 1% of the initial sample, were excluded because AMOS is unable to provide some fit indices when there are missing data). Maximum likelihood estimation was used for structural equation modeling and modification indices as well as a global appraisal of traditional indexes and their critical values were used, as suggested by Byrne (46), Tabachnick and Fidell (32), and Arbuckle (47): *CFI* (>0.90), *PCFI* (>0.75), GFI > 0.90 and *RMSEA* (<0.07). The statistical significance of the chi-square is reported but should not be used given the known limitation of this index with large samples (32, 46). *AIC* and *BIC* indices are reported to compare the models. The individual values of these indices are not meaningfully interpretable *per se*, as they are affected by sample size and other factors, but can be used to compared models estimated with the same sample (48). The smaller the value, the better. Differences in *AIC* values larger than 7 are considered very important, and differences larger than 10 suggest the model with the highest value is not supported (48).

To test the three-factor structure with five items associated to more than only one factor as proposed by Kennedy et al. (13), a confirmatory factor analysis was performed using AMOS 27.0 (47). The fit indices did not support the three-factor model proposed by Kennedy et al. (13): χ2 (96) = 823.23, *p* < 0.001, *CFI* = 0.84, *PCFI* = 0.67, *GFI* = 0.89, *RMSEA* = 0.09, *RMR* = 0.019. The *AIC* (903.23) and *BIC* (1094.25) for this model are reported to compare the three- and two-factor solutions (see below). Inadequate fit was also found when testing the model only with clinical or non-clinical participants (see Supplementary Material for the results), or after considering modification indices.

**Figure 1:**
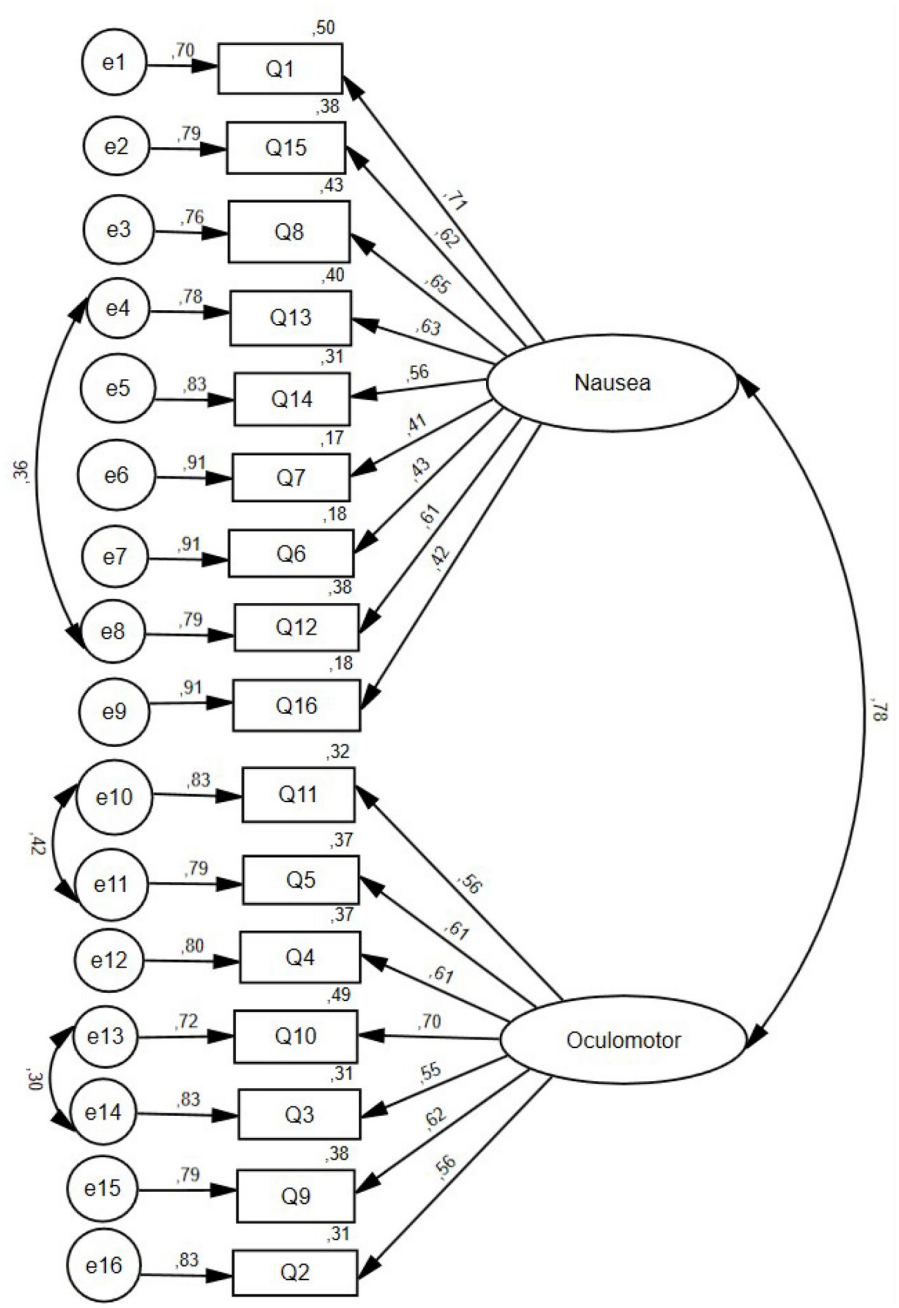
Final confirmatory factor analysis model of the *Simulator Sickness Questionnaire* with two factors observed in adults after an immersion in virtual reality.

To test the adequacy of the two-factor model proposed by Bouchard et al. (33) with items loading solely on one factor, a confirmatory factor analysis was performed using AMOS 27.0 (47), with maximum likelihood estimation. The final structural equation model is presented in Figure 1, where circles represent latent variable and rectangles represent measured variables (Q stands for Question or item number; e stands for error). The plausibility of the two-factor solution was confirmed by the fit indices (*CFI* = 0.89, *PCFI* = 0.74, *RMSEA* = 0.07, χ2 (100) = 578.15, *p* < 0.001), the examination of the modification indices, the low value of the *RMR* (0.016), and a strong percentage of variance explained (*GFI* = 0.92). Items with correlated errors are items assessing dizziness with eyes open and dizziness with eyes closed, difficulty focusing and blurred vision, and headache and fullness of head. A comparison between the two-factor and the original three-factor solutions confirmed that the two-factor solution was more parsimonious, based on the *AIC* (*AIC* = 650.15, vs. 903.23 for the 3-factor solution) and the *BIC* (*BIC* = 822.06 vs. 1094.25 for the 3-factor solution) criteria. Smaller *AIC* and *BIC* values indicate higher parsimony (32).

The mean *SSQ-Total raw* score in the current sample was 4.38 (*SD* = 5.07, range between 0 and 42). The mean subscale scores were 1.78 (*SD* = 2.79, range between 0 and 25) for the Nausea factor and 2.60 (*SD* = 2.88, range between 0 and 17) for the Oculomotor factor. The Cronbach's Alpha for the entire scale was 0.87.

In summary, the aims of Study 1 were to test if the three-factor structure of the *SSQ* found by Kennedy et al. (13) was replicable in a large sample of non-military adults immersed in VR and confirm if a two-factor structure was more adequate. The results indicated that Kennedy et al. (13) three-factor structure of the *SSQ* could not be replicated in a sample of non-military adults immersed in VR. Instead, they suggested that a two-factor structure is more parsimonious in this sample and favored a factor solution where each item loads only on one factor.

## Study 2

### Materials and Methods

The aim of Study 2 was to document the potential confound of symptoms caused by anxiety. The *SSQ* was administered before and after an anxiety-inducing task conducted *in vivo*, the Trier Social Stress Test in groups [*TSST*, (49) TSST-G, (50)], with no immersion in VR. The *TSST-G* is a validated standardized stressful procedure (49, 50). If the *SSQ* specifically measures unwanted negative side effects induced by immersions in VR, scores on the *SSQ* should not be significantly influenced by the *TSST-G* task nor correlate significantly with symptoms of anxiety.

#### Participants

A total of 91 adult (18 to 30 years old, *M* = 23.81, *SD* = 3.87) were recruited as part of other studies using the *TSST-G*. The first 42 participants were recruited for a study on facial expressions conducted by Daudelin-Peltier et al. (51). The remaining 49 participants were recruited for a subsequent study on visual perception from the same lab and matched for gender and age. In both studies, procedures regarding recruitment, sample characteristics, administration of the *SSQ*, and experimental manipulations with the *TSST-G* were identical. The methodology is reported in detail in Daudelin-Peltier et al. (51), and relevant information is summarized below. The final sample consists of 88 participants with no missing data on the *SSQ* (two participants did not show up and one participant had 43% of missing data).

#### Procedures

The UQO Research Ethics Board approved the project, participants provided their informed consent, and the *SSQ* was administered before and after the *TSST-G* (50) conducted *in vivo* (i.e., not in VR).

The *TSST* is essentially a social performance task to induce a subjective and physiological stress response (49). The three main components of the traditional *TSST* are: (1) an atmosphere of high performance, (2) the task induces socio-evaluative threat, and (3) participants have no control over the events (49, 50, 52). Participants completed a group *TSST-G* [i.e., three participants completed the tasks in turn in the same room with dividers separating them; see (51) for details]. Participants were called in front of an interview panel and had to: (a) give a speech on a topic outside of their comfort zone for three min per participant (for a total of nine min), and (b) perform an arithmetic task out loud for three min per participant (for a total of nine min). The two members of the interview panel displayed an emotionally neutral attitude and provided minimal feedback, except to ask participants to continue their presentation if they stopped before their three min and to start over the arithmetic task when they made an error. Anxiety and unwanted negative side effects induced by an immersion in VR were measured before and after the *TSST-G*.

To circumscribe the effect observed on the anxiety measure to the stressful nature of the *TSST-G*, as opposed to general characteristics of the *TSST-G* or the visit in the lab, a Control task was also used. In the Control task, participants were in the same room as for the *TSST-G*, read non-stressful magazines for nine min and counted out loud, starting from zero and at a pace of about one number per second, for three min each (for a total of nine min). The order in which participants completed the *TSST-G* and the Control task was randomly assigned. All measures were also administered before and after the Control task. Data for the Control task were analyzed only to confirm the experimental induction of anxiety with the *TSST-G*. The main analyses were performed using only *TSST-G* data.

#### Measures

In addition to the French version of the *SSQ* (see Study 1), participants completed the French version (53) of the State Anxiety scale from the *State-Trait Anxiety Inventory* (54). The State Anxiety scale of the *State-Trait Anxiety Inventory* consists of 20 items, each assessing how the participant feels right now on a scale from 1 (“*Almost Never*”) to 4 (“*Almost Always*”). After reverse scoring relevant items, all items are summed. A higher total score expresses more anxiety.

### Results

The statistical analyses were performed using SPSS 27.0 (55). When reporting total scores for the *SSQ*, the scoring procedure followed recommendations by Bouchard et al. (33): the total non-weighted score (i.e., *SSQ-Total* raw) was calculated by adding all 16 items of the *SSQ* only once and not multiplying the total by a constant. Prior to performing the analyses, assumptions were assessed using the SPSS Explore, Frequencies, and Regression functions for each of the variables used in further analyses. The assumption of normality was not met and there were univariate outliers. The univariate outliers (*z* = ± 3) were winsorized to the next most extreme score for the item in question. For example, if a participant had a *z*-score of 3 for one item with a corresponding raw score of 4, their raw score would be winsorized to the next most extreme raw score for that item (e.g., 3) by finding the next most extreme *z*-score (e.g., 2). Although this corrected extreme scores, the assumption of normality was still not met. As such, non-parametric analyses were performed with and without the univariate outliers, and the results did not significantly change. Therefore, the results of the non-parametric analyses with the univariate outliers not corrected (i.e., not winsorized) are presented here. Although there were multivariate outliers as shown by Mahalanobis distances above 20.09 [χ2 (8) = 20.09], they were included in the statistical analyses as our aim was to examine whether side effects induced without immersions in VR overlap with anxiety.

As a manipulation check, the impact of the Control task and the *TSST-G* on the State Anxiety scale were analyzed. In the Control task, a Wilcoxon signed-ranks test showed that participants' scores on the State Anxiety scale significantly decreased after completing the task [*Z*(88) = −2.40, *p* = 0.016], from a mean of 27.69 (*SD* = 6.76) to 26.52 (*SD* = 5.94). After participants performed the *TSST-G*, their scores significantly increased [*Z*(88) = 6.65, *p* < 0.001], from a mean of 27.11 (*SD* = 6.16) to 35.22 (*SD* = 12.18), indicating that the procedure successfully induced anxiety. For the *SSQ-Total raw* scores in the Control condition, a Wilcoxon signed-ranks test indicated that participants' scores significantly increased after completing the task [*Z*(87) = 4.09, *p* < 0.001], from a mean of 2.86 (*SD* = 2.67) to 4.30 (*SD* = 3.49).

#### Overlap With Anxiety

To examine the relationship between anxiety and symptoms measured by the *SSQ*, the Spearman correlation between the total state anxiety post-*TSST-G* and items of the *SSQ* post-*TSST-G* is reported in Table 1. Results revealed that only five *SSQ* items did *not* significantly correlate with anxiety post-*TSST-G*. The eleven items that significantly correlated with anxiety were evenly distributed between the Nausea (items 1, 6, 7, 8, 14, and 15) and Oculomotor (items 5, 9, 10, 11, and 12) factors. After applying a Bonferroni correction (0.05/16 = 0.003), correlations for items number 1, 5, 7, 9 and 10 remain statistically significant.

**Table 1:** Correlation of *SSQ* items with the State Anxiety scale.

***SSQ*after the *TSST-G***	**State Anxiety after the *TSST-G***
Item 1 - General discomfort	0.67***
Item 2 - Fatigue	0.11
Item 3 - Headache	−0.07
Item 4 - Eyestrain	0.15
Item 5 - Difficulty focusing	0.45***
Item 6 - Increased salivation	0.22*
Item 7 - Sweating	0.40***
Item 8 - Nausea	0.25*
Item 9 - Difficulty concentrating	0.49***
Item 10 - Fullness of head	0.48***
Item 11 - Blurred vision	0.29**
Item 12 - Dizzy (eyes open)	0.26*
Item 13 - Dizzy (eyes closed)	0.09
Item 14 - Vertigo	0.28**
Item 15 - Stomach awareness	0.24*
Item 16 - Burping	0.130
*SSQ-Total* Raw	0.60***

*N = 88*.

*p < 0.05, **p < 0.01, ***p < 0.001.

Another statistical approach to address the overlap between *SSQ* scores and anxiety is to perform a factor analysis to see how items aggregate on common or distinct factors. The reasoning behind the statistical analyses was that the items of the State Anxiety scale and the *SSQ* should theoretically be orthogonal and load on two distinct factors. Items of the *SSQ* significantly loading on the anxiety factor would show that these symptoms were associated with anxiety, or at least not specifically representing side effects of immersions in VR. A principal component analysis with a Varimax rotation was conducted on all post-*TSST-G* items of the State Anxiety scale and the *SSQ* to determine the overlap between anxiety and VR-induced side effects. The Kaiser-Meyer-Olkin measure of sampling adequacy was.82 and Bartlett's test of sphericity was significant [χ2 (630) = 2024.55, *p* < 0.001], suggesting that it was appropriate to conduct a factor analysis on this dataset. The ratio of participants per variable was low (two participants per variable), which led to the decision to be conservative and consider only loadings higher than 0.50 as significant. The number of factors to extract was forced to two, with the expectation that items of each questionnaire would load on their respective factor with minimal cross-loadings. The eigenvalues of the first and second factors were 12.59 (34.97% of unique variance) and 2.91 (8.08% of unique variance), followed by six other potential factors with eigenvalues greater than one and accounting for a portion of unique variance ranging from 6.28% to 3.01%. Examination of the rotated loading matrix revealed that two items of the *SSQ* loaded above 0.50 on the State Anxiety factor: (1) item 1– General discomfort (cross-loading = 0.69), and (2) item 9 – Difficulty concentrating (cross-loading = 0.53). Three additional items had loadings approaching 0.50 on the State Anxiety factor: item 7 – Sweating (cross-loading = 0.46), item 8 – Nausea (cross-loading = 0.48), and item 14 – Vertigo (cross-loading = 0.49). All these loadings were stronger on the anxiety factor than on the *SSQ* factor (item 9 had a loading of 0.43 on the *SSQ* factor) and evenly distributed between the Nausea and Oculomotor factors reported in Study 1.

#### Overlap With Increase in Anxiety

The intensity of symptoms assessed by the *SSQ-Total raw* was low before the *TSST-G*, with a mean of 2.98 (*SD* = 2.73), and significantly increased [*Z*(88) = 3.96, *p* < 0.001] to 5.01 (*SD* = 4.51) post-*TSST-G*. Wilcoxon signed-ranks tests were conducted for all *SSQ* items pre- and post-*TSST-G* to examine which ones had significantly increased after performing the *TSST-G in vivo*. The results indicated that items number 1 [*Z*(88) = 5.02, *p* < 0.001], number 3 [*Z*(88) = 2.32, *p* = 0.020], number 5 [*Z*(88) = 3.98, *p* < 0.001], number 7 [*Z*(88) = 2.91, *p* = 0.004], number 9 [*Z*(88) = 4.48, *p* < 0.001], number 10 [*Z*(88) = 2.04, *p* = 0.042], and number 11 [*Z*(88) = 2.33, *p* = 0.020] significantly increased post-*TSST-G*. After applying a Bonferroni correction (0.05/16 = 0.003), increases on items 1, 5, and 9 remained statistically significant.

To analyze increase in anxiety and increase in *SSQ* items while maintaining sufficient statistical power, the next statistical analysis focused on the seven symptoms measured by the *SSQ* that changed following the *TSST-G*. Standardized pre-post *TSST-G* residuals for each item and for the State Anxiety scale were used as change scores. A multiple regression was performed with the residualized change pre/post *TSST-G* State Anxiety scale as a dependent variable and the seven residualized change pre/post *TSST-G SSQ* scores as predictors. The assumptions of linearity, absence of multicollinearity (Tolerance and VIF statistics), homoscedasticity, and independence of the residuals (Durbin-Watson statistic) were met. No influential cases biasing the model were found (Cook's Distance values were all under 1). The seven-predictor model accounted for 51% of the variance in change in anxiety [*F*(7, 80) = 12.09, *p* < 0.001, adj*R*^2^ = 0.47]. Results for each item are reported in Table 2. Only change in general discomfort (*SSQ* item 1) and difficulty concentrating (*SSQ* item 9) made a significant unique contribution to the change in State Anxiety following the *TSST-G*. Interestingly, performing the same analysis to predict scores of anxiety post *TSST-G*, instead of increase in anxiety, revealed that item #7 became a significant predictor (β = 0.17, *t* = 2.00, *p* = 0.049, *sr* = 0.15) and item #9 became non-significant (β = 0.18, *t* = 1.72, *p* = 0.090, *sr* = 0.13).

**Table 2:** Multiple regression predicting (residualized) increase in State Anxiety score with (residualized) change observed on selected items of the *SSQ* before and after a *TSST-G* conducted *in vivo*.

***SSQ* Items (residualized change scores)**	**β**	***t***	***p***	***sr***	***95% Confidence intervals***	
Item 1 – General Discomfort	0.45	4.53	0.000	0.35	0.25	0.65
Item 3 – Headache	−0.10	−1.20	0.235	−0.09	−0.26	0.07
Item 5 – Difficulty focusing	0.15	1.36	0.179	0.11	−0.07	0.38
Item 7 – Sweating	0.11	1.23	0.222	0.10	−0.07	0.30
Item 9 – Difficulty concentrating	0.24	2.21	0.030	0.17	0.02	0.46
Item 10 – Fullness of head	−0.05	−0.50	0.620	−0.04	−0.27	0.16
Item 11 – Blurred vision	−0.08	−0.74	0.461	−0.06	−0.28	0.13

*N = 88*.

In summary, Study 2 documented the potential confound of symptoms caused by anxiety when assessing unwanted negative side effects of immersions in VR. A variety of statistical strategies were used, and two items systematically stand out as potentially problematic. General discomfort and difficulty concentrating can very well convey the notion of “cybersickness”, but they are also very likely to be influenced by anxiety. The item measuring sweating was also recurrently highlighted as influenced by anxiety but predicted an *increase* in anxiety to a lesser extent than other items.

## Discussion

Immersions in VR can lead to unwanted side effects and, as the applications of this technology grow, the need to monitor their symptoms is important. The most popular self-administered tool to assess unwanted negative side effects following immersions in VR is the *SSQ* (13). This tool has a very strong track record and is practical for professionals using VR for mental health applications, among others. However, simulator sickness (i.e., induced by the actual movement of the simulator) and VR-induced side effects (i.e., induced by visual information without matching vestibular information and specifically experienced in VR) are relatively distinct phenomena (56). As such, using the *SSQ* for immersions in VR, as opposed to flight simulators, and with users from the general population has raised a few questions in recent years e.g., (57). One of these questions is whether the symptoms measured by the *SSQ* measure two distinct latent dimensions [a Nausea factor and an Oculomotor factor; (33)] or also additional dimensions such as disorientation (13). A second question is whether anxiety induced by the immersion may contaminate symptoms measured by the *SSQ* in applications in the treatment of anxiety and other psychological disorders [e.g., chronic pain, (42)]. If this is the case, some symptoms should not be attributed to VR technology, but as a normal consequence of the therapeutic use of VR, such as *in virtuo* exposure for anxiety disorders (58), relaxation in VR, or vestibular rehabilitation e.g., (59).

Results from Study 1 do not support the three-factor model proposed by Kennedy et al. (13). Results of confirmatory factor analyses clearly revealed that, with a sample of civilians immersed in a wide variety of technologies, the *SSQ* essentially measures two distinct latent dimensions: nausea symptoms and oculomotor symptoms. This finding has practical implications for scoring the *SSQ*. According to Kennedy et al. (13), the total score is calculated after adding the score of the items from each factor, summing the three factors, and multiplying the result by a constant. Since five items (i.e., 1, 5, 8, 9, 11) load on more than one of the three factors, these items have twice the weight on the total score than the others and the latent dimensions of the *SSQ* overlap with each other. As mentioned in (33, 35), this scoring method is not intuitive, and many researchers do not report if they followed it or not when calculating the total score of the *SSQ*. Recommendations about the scoring of the *SSQ* will be summarized in the Conclusion, but arguments are strong in favor of considering that the *SSQ* comprises only two factors. At face value, it is surprising to allocate symptoms of nausea to a Disorientation factor when it is the defining feature of the Nausea factor, or to allocate blurred vision to the Disorientation factor when it is also in the Oculomotor factor. Empirical analyses reported previously (33) and in Study 1 strongly support the relevance of Nausea and Oculomotor dimensions found by Kennedy et al. (13), but not the model that includes a Disorientation factor. Differences in applications and populations may explain the difference in factor structures. The *SSQ* was meant to be used with training simulators with physically fit military personnel, not for immersions in VR with users from the general population. As pointed out by Lawson et al. (3), military personnel may be less likely to experience negative side effects since they are more likely to be frequently involved in challenging vehicle motion, in better physical shape, or able to remain immersed in VR longer despite feeling unwanted effects. Stanney et al. (56) also suggested that negative side effects differ between immersion in VR and in training simulators. The design of the VE and the task performed are known to have an impact on the induction of negative side effects (3, 19, 60). Replications of our results in other centers, with diverse populations and methodologies, remain warranted.

Results from Study 2 confirm the strong correlation between scores on the *SSQ* and anxiety, but most importantly, they show a significant association between anxiety and many symptoms measured by the *SSQ* despite the fact that symptoms were not induced by an immersion in VR. Only five symptoms did not significantly correlate with anxiety after a task performed *in vivo*: fatigue, headache, eyestrain, dizziness when eyes are closed, and burping. If one wants items likely to specifically measure unwanted negative side effects of an immersion in VR, these five items are the most likely candidates. Other items of the *SSQ* that correlate with anxiety are not to be discarded too rapidly, as various analytical approaches in Study 2 yielded slightly different conclusions. Study 2 examined increases on each item after a stressful task conducted *in vivo* and, after controlling for the number of comparisons, items measuring general discomfort and difficulty concentrating clearly increased when they should not. These two items were also among those loading strongly on the anxiety factor constituted by all items of the State Anxiety scale. A multiple regression supported the finding while controlling for shared variance. The *SSQ* item measuring sweating was correlated with anxiety after the stressful task conducted *in vivo*, but not with increase in anxiety. Other items may seem problematic in some analyses but not in others.

### Limitations

The methodologies used in the article are not without limitations. The sample used to examine the factor structure of the *SSQ* is heterogeneous. Participants vary in age, gender, the presence or absence of anxiety disorders (see Supplementary Material for analyses performed separately), tasks performed in VR, hardware, and software. We consider such a variety a strength in terms of external validity and potential for generalization of the results. However, the consequence is the impossibility to isolate specific factors that can influence the findings. As the majority of the sample is aged between 20 and 50, results may be less relevant for children, adolescents and older adults. Replications are therefore required. We encourage researchers to publish factor analyses on samples that may limit the generalization of results but would provide information specific to populations, tasks, and hardware. A systematic approach examining human factors e.g., (1, 7, 19) is strongly recommended. Documenting the factor structure of the *SSQ* with children and older adults should also be done.

It is possible to criticize Study 2 by arguing that testing the *SSQ* without an immersion in VR is irrelevant because the *SSQ* was meant to be used in the context of immersions in VR or with simulators. However, the correlation between anxiety and unwanted side effects of immersions in VR had already been documented by several independent groups after immersions in VR e.g., (35–37, 39–41). Documenting symptoms that should be induced by immersions in VR occurring in contexts where there is no VR challenges the construct validity of the instrument, or at least some of its items. The ideal study should: (a) be designed exclusively for the purpose of documenting the potential overlap between unwanted side effects of immersions in VR and anxiety; (b) have participants perform an anxiety induction task such as the *TSST, in vivo*, in VR, and in a control condition to isolate all potential confounds; (d) have a sample large enough to benefit from sufficient statistical power and variance to compare pre/post residualized changes among the three experimental conditions; and (e) include participants with and without an anxiety disorder, or other relevant clinical conditions such as addictions or pain, for example. The statistical approaches for Study 2 can also be criticized as somewhat redundant because the analyses all represent variations on the general lineal model (31, 32). Indeed, Study 2 was based on an exploratory approach. Based on the findings from this article and the methodological suggestions above, it is our hope that new studies will be designed to test specific hypotheses emerging from our results.

As summarized below, our recommendation is not to systematically remove the two items that are identified as symptoms of anxiety. This can be criticized as a weak recommendation. Why not remove these items altogether and avoid potential confusion? These two items may measure lighter or early symptoms of immersions in VR. Also, just as fever is a common symptom of many medical disorders, these symptoms may target common factors between anxiety and unwanted side effects of immersions in VR. General discomfort and difficulty concentrating may be relevant to describe unwanted side effects of immersions in VR, but they are not defining features. When it is important to measure pathognomonic symptoms and avoid false positives, it is better not to include these symptoms. It is prudent to administer all 16 items of the *SSQ* until the relevance of these two symptoms has been characterized and examined in other contexts.

### Conclusion

Considering the findings of these two studies, there is a need to revise the *SSQ* when assessing unwanted negative side effects following an immersion in VR. At the same time, it is important to avoid confusion in the field and to facilitate comparisons between studies and samples. We propose the following recommendations for people interested in measuring unwanted negative side effects in users of VR from the general population: (a) administer all 16 original items of the *SSQ* from Kennedy et al. (13), before and after immersions in VR; (b) report *SSQ-Total raw* scores by adding the scores from all items, without giving more weight to some items than others; (c) conceptualize the *SSQ* as an instrument with a two-factor latent structure, with a Nausea factor (items 1, 6, 7, 8, 12, 13, 14, 15, and 16) and an Oculomotor factor (items 2, 3, 4, 5, 9, 10, and 11); and (d) when immersions involve reporting anxiety or other clinical conditions report, in addition to the above information, *SSQ-Total-Anx, SSQ-Nausea-Anx*, and *SSQ-Oculomotor-Anx* scores that are scored without items 1 and 9 due to their important overlap with anxiety. We also recommend that researchers independently replicate our results with different populations (e.g., people with addictions, schizophrenia, acute or chronic pain) and continue documenting the relevance of the item measuring sweating.

The findings about the overlap with some symptoms of anxiety must raise the awareness of experimenters, clinicians, and users about the fact that they should not automatically attribute post-immersion symptoms to VR, particularly if users are exposed to an anxiety-inducing VE. To ensure public safety, unwanted negative side effects should be systematically monitored before, during, and after immersions when researchers are developing new VR tools and treatments. A detailed usage protocol to inform users and reduce the likelihood of unwanted side effects can be found in Stanney et al. (61) to guide professionals using VR in mental health applications.

## Data Availability Statement

The raw data supporting the conclusions of this manuscript will be made available by the authors, without undue reservation, to qualified researchers and for audits.

## Ethics Statement

The studies involving human participants were reviewed and approved by the Comité d'éthique de la recherche de l'Université du Québec en Outaouais. The patients/participants provided their written informed consent to participate in this study.

## Author Contributions

SB contributed to the literature review, the methodology, data collection and statistical analyses, and writing and revising the manuscript. MB contributed to the literature review, statistical analyses, and writing and revising the manuscript. GR contributed to the methodology, data collection, and revising the manuscript. PR contributed to the methodology and data collection of Study 1 and revising the manuscript. HF, CD-P, CB, and DF contributed to the methodology and data collection of Study 2 and revising the manuscript. All authors contributed to the article and approved the submitted version.

## Funding

This project was supported by grants from the Canada Research Chairs (#950-231039 and #950-210762), the Natural Science and Engineering Research Council of Canada (#50262982), and the Social Sciences and Humanities Research Council of Canada (#305205).

## Conflict of Interest

SB is the President of, and owns equity in, *Cliniques et Développement In Virtuo*, a spin-off from the university that uses virtual reality and distributes virtual environments. In addition, GR is the Vice-President of Corporate Affairs of, and owns equity in, *Cliniques et Développement In Virtuo*. The terms of these arrangements have been reviewed and approved by the Université du Québec en Outaouais in accordance with its conflict of interest policies. The remaining authors declare that the research was conducted in the absence of any commercial or financial relationships that could be construed as a potential conflict of interest.

## Publisher's Note

All claims expressed in this article are solely those of the authors and do not necessarily represent those of their affiliated organizations, or those of the publisher, the editors and the reviewers. Any product that may be evaluated in this article, or claim that may be made by its manufacturer, is not guaranteed or endorsed by the publisher.

## Abbreviations

HMD: Head mounted display
SSQ: Simulator Sickness Questionnaire
TSST: Trier Stress Social Test
TSST-G: Trier Stress Social Test in Groups
VE: Virtual environment
VR: Virtual reality.
