# Supplementary material for: Arguing in Favor of Revising the Simulator Sickness Questionnaire Factor Structure When Assessing Side Effects Induced by Immersions in Virtual Reality
Source: Front Psychiatry. 2021 Nov 5;12:739742. doi: 10.3389/fpsyt.2021.739742 (PMC8604025; doi:10.3389/fpsyt.2021.739742)
Supplement: Supplementary file 1 [file Data_Sheet_1.docx]

Supplementary Material

Testing the Two-Factor Structure of the *Simulator Sickness Questionnaire* in Clinical and Non-Clinical Participants

# Supplementary Data

Because we were interested in the use of the *Simulator Sickness Questionnaire* (*SSQ*) in clinical and non-clinical samples, the main article examined the two- and three-factor structures of the *SSQ* by combining all participants in Study 1. In this online supplement, we present the results of the confirmatory factor analyses conducted separately for the non-clinical and the clinical participants from Study 1.

The clinical sample consisted of 423 adults (306 women, 117 men) recruited from the general population and diagnosed with an anxiety disorder (*n* = 346) or gambling disorder (*n* = 77). The non-clinical sample (i.e., healthy controls) comprised 453 adults (245 women, 207 men) who did not receive these diagnoses. The mean age of the clinical and non-clinical participants was 37.90 (*SD* = 13.73, range between 16 to 76) and 32.06 (*SD* = 12.17, range between 15 to 76), respectively. Eight clinical participants and one non-clinical participant with missing data were excluded from the analyses. The ratio of participants per variable was 26 to 1 (clinical sample) and 28 to 1 (non-clinical sample), confirming that the samples met basic assumptions and criteria to perform a factor analysis (Nunnally, 1978).

To calculate the *SSQ-Total raw* score, the non-weighted procedure was followed (i.e., summing all raw 16 items of the *SSQ* only once and not multiplying the total by a constant). The mean *SSQ-Total raw* score, Nausea score, and Oculomotor score in the non-clinical sample were 2.96 (*SD* = 3.86, range between 0 and 42), 1.09 (*SD* = 2.15, range between 0 and 25), and 1.87 (*SD* = 2.29, range between 0 and 17), respectively. The mean *SSQ-Total raw* score*,* Nausea score, and Oculomotor score in the clinical sample were 5.90 (*SD* = 5.73, range between 0 and 26), 2.52 (*SD* = 3.18, range between 0 and 17), and 3.38 (*SD* = 3.22, range between 0 and 15), respectively. The mean *SSQ-Total-Anx, SSQ-Nausea-Anx*, and *SSQ-Oculomotor-Anx* in the clinical sample were 4.84 (*SD* = 4.84, range between 0 and 22), 1.89 (*SD* = 2.67, range between 0 and 14) and 2.94 (*SD* = 2.84, range between 0 and 13), respectively.

Maximum likelihood estimation was used for structural equation modeling and modification indices as well as a global appraisal of traditional indexes and their critical values were used, as suggested by Byrne (1994), Tabachnick and Fidell (2007), and Arbuckle (2020): CFI (> .90), PCFI (> .75), GFI > .90 and RMSEA (< .07). The statistical significance of the chi-square is reported but should not be used given the known limitation of this index with large samples (Byrne, 1994; Tabachnick & Fidell, 2007).

The results of confirmatory factor analyses testing Kennedy et al.’s (1993) three-factor structure indicated that it was inadequate for both the non-clinical [χ2 (96) = 499.95, *p* < .001, *CFI* = .81, *PCFI* = .65, *GFI* = .89, *RMSEA* = .10, *RMR* = .015, *AIC* = 579.95, *BIC* = 744.58] and clinical [χ2 (96) = 501.46, *p* < .001, *CFI* = .81, *PCFI* = .65, *GFI* = .88, *RMSEA* = .10, *RMR* = .028, *AIC* = 581.46, *BIC* = 743.36] samples.

For the non-clinical sample, the plausibility of the two-factor solution was confirmed by the fit indices (*CFI* = .90, *PCFI* = .75, *RMSEA* = .07, *AIC* = 391.34, *BIC* = 539.51), the examination of the modification indices, the low value of the *RMR* (.011), and a strong percentage of variance explained (*GFI* = .92). The chi-square was also significant, χ2 (100) = 319.34, *p* < .001. The plausibility of the two-factor solution was also confirmed for the clinical sample, as shown by the fit indices much closer to the critical values than the alternative model (*CFI* = .85, *PCFI* = .71, *RMSEA* = .09, *AIC* = 497.86, *BIC* = 643.56), the examination of the modification indices, the low value of the *RMR* (.026), and a strong percentage of variance explained (*GFI* = .89). The chi-square was significant, χ2 (100) = 425.86, *p* < .001. It is important to note that although some of the fit indices reached the critical values for the three-factor solution (e.g., *GFI* = .89), a larger number of fit indices reached the critical values for the two-factor solution in both the non-clinical and clinical samples. This conclusion is also clearly supported when comparing the AIC and BIC indices (Burnham & Anderson, 2004). The final structural equation models are presented in Supplementary Figure 1 and Supplementary Figure 2, where circles represent latent variable and rectangles represent measured variables (Q stands for Question or item number; e stands for error). Finally, the plausibility of the two-factor solution without items 1 and 9 was tested with the clinical sample. The results seemed to favor this structure based on the fit indices (*CFI* = .86, *PCFI* = .69, *RMSEA* = .09, *AIC* = 379.74, *BIC* = 509.26), the examination of the modification indices, the low value of the *RMR* (.024), and a strong percentage of variance explained (*GFI* = .91). The chi-square was significant, χ2 (73) = 315.74, *p* < .001. In short, the fit indices favored the two-factor model, and thus, suggest that it is more appropriate in both clinical and non-clinical populations. As discussed in the article, replications with less heterogenous clinical samples may lead to improved fit.

# Supplementary Figures

**
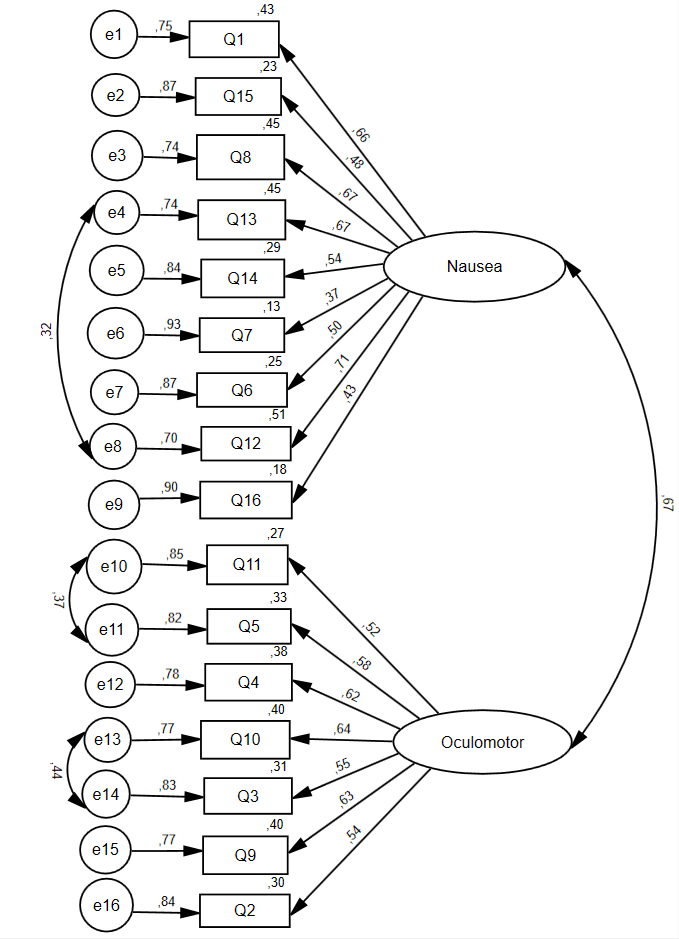
**

**Supplementary Figure 1.** Confirmatory factor analysis of the *SSQ* with two factors in non-clinical participants

**
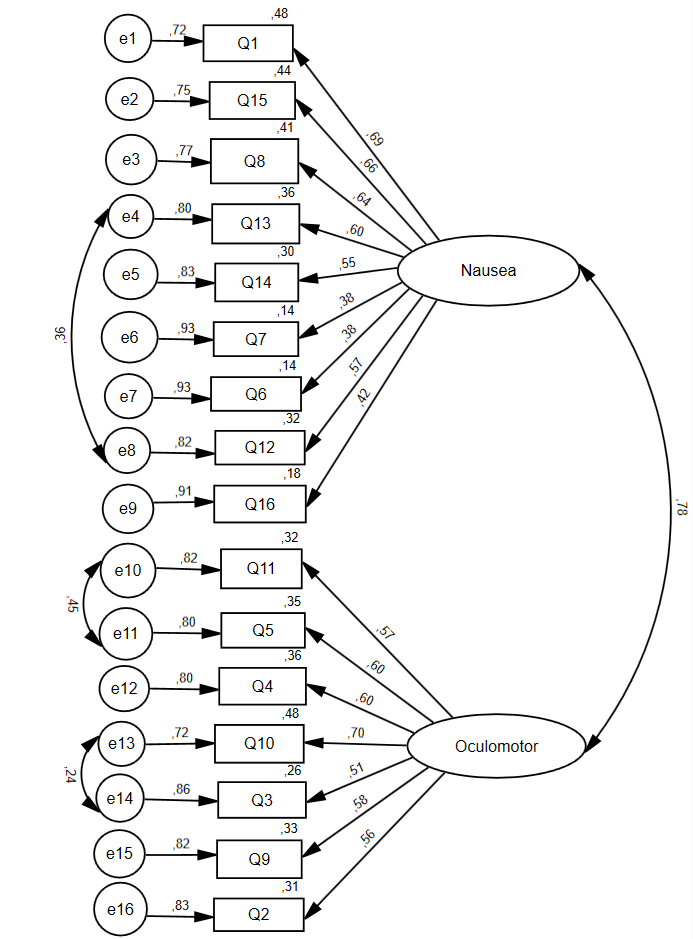
**

**Supplementary Figure 2.** Confirmatory factor analysis of the *SSQ* with two factors in clinical participants

**Reference of studies used for the sample in Study 1**

The sample for Study 1 consists of data collected in the following studies.

Aimé, A., Cotton, K., & Bouchard, S. (2009). Reactivity to virtual reality immersions in a subclinical sample of women concerned with their weight and shape. *Journal of Cybertherapy and Rehabilitation*, 2(2), 111-120.

Baus, O., Bouchard, S., Gougeon, V., & Roucaut, F.-X. (2011). Comparison of anxiety in response to virtual spiders while immersed in augmented reality, head-mounted display, or cave-like system. Oral presentation at the 1*6th Annual Cybertherapy & Cyberpsychology Conferenc*e, Gatineau, June 20-22.

Bouchard, S., Bernier, F., Boivin, É., Dumoulin, S., Laforest, M., Guitard, T., et al. (2013). Empathy toward virtual humans depicting a known or unknown person expressing pain. Journal of *CyberPsychology, Behavior and Social Networking*, 16(1), 61-71. DOI: 10.1089/cyber.2012.1571

Bouchard, S., Bernier, F., Boivin, E., Guitard, T., Laforest, M. Dumoulin, S. & Robillard, G. (2012). Modes of immersion and stress induced by commercial (off-the-shelf) 3D games. *Journal of Defense Modeling and Simulation: Applications, Methodology, Technology,* 1-14. DOI: 10.1177/1548512912446359.

Bouchard, S., Bernier, F., Boivin, E., Morin, B., & Robillard, G. (2012). Using biofeedback while immersed in a stressful videogame increases the effectiveness of stress management training. *PLoS ONE*, 4(7), 1-11. DOI: 10.1371/journal.pone.0036169.

Bouchard, S., Côté, S., Robillard, G., St-Jacques, J., & Renaud, P. (2006). Effectiveness of virtual reality exposure in the treatment of arachnophobia using 3D games. *Technology and Health Care,* 14(1), 19-27. DOI: 10.3233/THC-2006-14103

Bouchard, S., Dumoulin, S., Labonté-Chartrand, G., & Robillard, G., & Renaud, P. (2006). Perceived realism has a significant impact on the feeling of presence. Oral presentation at the *11th Annual CyberTherapy Conference 2006*, Gatineau (Québec), June 12-15.

Bouchard, S., Dumoulin, S., Robillard, G., Guitard, T., Klinger, É., Forget, H., Loranger, C., & Roucaut, F.-X. (2017). Exposure in virtual reality is more effective and efficient than in vivo exposure when using broad range of stimuli for the treatment of social anxiety disorder: A three-arm randomized controlled trial. *British Journal of Psychiatry,* 210(4), 276-283. DOI: 10.1192/bjp.bp.116.184234

Bouchard, S., & Labonté-Chartrand, G. (2011). Emotions and the emotional valence afforded by the virtual environments. In J.-J. Kim (Ed.) *Virtual Reality* (pp. 501-514). Croatia: InTech.

Bouchard, S. Robillard, G., Giroux, I., Jacques, C., Loranger, C., St-Pierre, M., Chrétien, M., & Goulet, A. (2017). Using virtual reality in the treatment of gambling disorder: The development of a new tool to practice cognitive behaviour therapy. *Frontiers in Psychiatry*, 8(27), 10pp. DOI: 10.3389/fpsyt.2017.00027

Bouchard, S., St-Jacques, J., Renaud, P., & Wiederhold, B.K. (2009). Side effects of immersions in virtual reality for people suffering from anxiety disorders. *Journal of Cybertherapy and Rehabilitation*, 2(2), 127-137.

Bouchard, S., St-Jacques, J., Robillard, G., & Renaud, P. (2008). Anxiety increases the sense of presence in virtual reality. *Presence: Teleoperators and Virtual Environments*, 4(1), 376-391. DOI: 10.1162/pres.17.4.376

Côté, S. & Bouchard, S. (2005). Documenting the efficacy of virtual reality exposure with psychophysiological and information processing measures. *Applied Psychophysiology and Biofeedback,* 30(3), 217-232. DOI: 10.1007/s10484-005-6379-x

Dumoulin, S., Gougeon, V., Loranger, C., & Bouchard, S. (2011). Are distraction and attention the main variables in pain management: An experimental study using a cold-pressor test and virtual reality. Poster presentation at the *45th Annual Convention of the Association for Behavioral and Cognitive Therapy (ABCT)*, Toronto, November 10-13.

Guitard, T., & Bouchard, S. (2006). Failure to influence presence by manipulating narrative content. Poster presentation at the *11th Annual CyberTherapy Conference 2006,* Gatineau (Québec), June 12-15.

Guitard, T., Bouchard, S., Bélanger, C., & Berthiaume, M. (2019). Exposure to a standardized catastrophic scenario in virtual reality on a personalized scenario in imagination for generalized anxiety disorder. *Journal of Clinical Medecine*, 8(309), DOI:10.3390/jcm8030309

Hajjar, A., Bouchard, S., Loranger, C., & Berthiaume, M. (2020). The efficacy of performing tasks in virtual reality that are infeasible in vivo: a clinical trial with acrophobia sufferers. *Cypsy25 Conference.*

Laforest, M., Bouchard, S., Crétu, A.-M., & Mesly, O. (2016). Inducing an anxiety response using a contaminated virtual environment: Validation of a therapeutic tool for obsessive-compulsive disorder. *Frontiers in ICT*, 3(18), 1-11. doi: 10.3389/fict.2016.00018.

Michaud, M., Bouchard, S., Dumoulin, S., & Zhong, X.-W. (2004). Manipulating presence and its impact on anxiety. Poster presentation at the *Cybertherapy Conference 2004*, San Diego, January 10-12.

Quintana, P., Nolet, K., Baus, O., & Bouchard, S. (2019). The effect of exposure to fear-related body odorants on anxiety and interpersonal trust toward a virtual character. *Chemical Senses*, 44(9), 683-692. DOI: 10.1093/chemse/bjz063

Robillard, G., Bouchard, S., Fournier, T., & Renaud, P. (2003). Anxiety and presence during VR immersion: A comparative study of the reactions of phobic and non-phobic participants in therapeutic virtual environments derived from computer games. *CyberPsychology and Behavior*, 6(5), 467-476. DOI: 10.1089/109493103769710497

Tardif, N., Therrien, C.-É., Bouchard, S. (2019). Re-examining psychological mechanisms underlying virtual reality-based exposure for spider phobia. *Cyberpsychology, Behavior, and Social Networking,* 22(1), 39-45. DOI: 10.1089/cyber.2017.0711
